# Recent advances on gradient hydrogels in biomimetic cartilage tissue engineering

**DOI:** 10.12688/f1000research.12391.2

**Published:** 2018-06-28

**Authors:** Ivana Gadjanski

**Affiliations:** 1 Belgrade Metropolitan University, Belgrade, Serbia; 2 BioSense Institute, University of Novi Sad, Novi Sad, Serbia

**Keywords:** articular cartilage, tissue engineering, gradient hydrogels

## Abstract

Articular cartilage (AC) is a seemingly simple tissue that has only one type of constituting cell and no blood vessels and nerves. In the early days of tissue engineering, cartilage appeared to be an easy and promising target for reconstruction and this was especially motivating because of widespread AC pathologies such as osteoarthritis and frequent sports-induced injuries. However, AC has proven to be anything but simple. Recreating the varying ****properties of its zonal structure is a challenge that has not yet been fully answered. This caused the shift in tissue engineering strategies toward bioinspired or biomimetic approaches that attempt to mimic and simulate as much as possible the structure and function of the native tissues. Hydrogels, particularly gradient hydrogels, have shown great potential as components of the biomimetic engineering of the cartilaginous tissue.

## Introduction

Cartilage and osteochondral (OC) tissue engineering (TE) has two aims: it attempts to (1) reconstruct and repair disturbed cartilage and underlying bone tissue, ideally making the functional OC complex, comprised of cartilage/bone phases connected through the OC interface and (2) generate new OC tissues that can be used for disease modeling and drug discovery ^1^. However, there are a number of challenges in this process and this is primarily due to the very specific characteristics of the cartilage, its zonal structure, and still not fully defined properties of the cartilage-bone interface. The functional properties of cartilage and the cartilage-bone interface (in the context of the zones) are particularly challenging to achieve; if the function is not fully restored in the engineered tissue with the appropriate zones, then the new tissue is at risk of being damaged. Thus, there is a need to develop new strategies to help direct cells to form the zones of the tissue.

Use of gradient hydrogels, biomaterials with spatiotemporally varying physical and chemical properties, has proven to be one of the most promising approaches and is in line with the biomimetic strategy in TE. This review will focus on recent advances in the use of gradient hydrogels in cartilage TE. However, it should be emphasized that implementation of biomimetic hydrogels is a fast-growing field and has great potential for clinical application. It is nearly impossible to give a concise yet thorough review of such an expanding field. Hence, the readers are invited to regard the provided information as the concise overview of some of the most innovative recent approaches in the fabrication and implementation of gradient hydrogels for cartilage TE.

## Why is cartilage difficult to repair and reconstruct?

Cartilage heals very poorly on its own and this is primarily due to its avascular nature. It has a low number of constituting cells—chondrocytes—which make up only 5% of total cartilage volume ^2^ and are enclosed in a unique cartilaginous extracellular matrix (ECM), made of collagen fibers pre-stressed by osmotic swelling pressure exerted by negatively charged proteoglycans embedded in the collagen network ^3^. ECM makes up about 25% of the total volume of cartilage and is a dynamic structure, providing mechanical support as well as growth factors and cytokines secreted by the cells. The rest of the roughly 70% of the wet weight of cartilage tissue is attributable to water, which is responsible for cartilage’s unique biomechanics, flexibility, reversible deformation ability, and strength ^4^. Owing to its avascular nature, native cartilage is hypoxic and has a spatial oxygen gradient of less than 1% in the deepest zones and up to less than 10% at the surface ^5, 6^. Zonality of the cartilage is reflected in both the chondrocyte morphology and the ECM organization (collagen type II decreases from the superficial zone to the deep calcified cartilage, while the levels of collagen type X, proteoglycans, and sulphated glycosaminoglycans (sGAGs) increase ^2^), which has been very difficult to achieve in the engineered constructs. The cartilage-bone interface is another feature of the OC complex that has been notoriously challenging to engineer. These challenges reflect the very gist of the biomimetic approach in TE, which can be summarized as the attempt to generate a tissue construct with structure and function as similar as possible to those of the native, biological counterpart.

One of the best strategies for achieving biomimetic zonality in the engineered cartilage tissue is to use gradient hydrogels, which can provide spatiotemporal gradients of multiple physical cues (for example, ECM stiffness and porosity) and biochemical cues (for example, morphogens) ^1^. As for the bone phase in the OC construct, hydrogels are not the primary choice and this is due to their low mechanical stiffness ^7^; hybrid scaffolds—hydrogels for the cartilaginous phase and rigid, porous scaffolds for the bone region—are used instead ^2, 8^. The cellular components of the construct comprise either primary cells (chondrocytes for the cartilaginous phase and osteoprogenitors for the osseous part) or stem/stromal cells, preferably autologous, that can be isolated from various tissues induced to chondrogenesis/osteogenesis ^9^. Mesenchymal stromal/stem cells (SCs) are currently viewed as the cell type of choice—particularly adipose-derived cells because of the relative ease of extraction from the fat tissue—for potential clinical treatments ^10^. There are two modes of cell integration to hydrogels: cells seeded on the hydrogel surface (2D approach) or encapsulation of cells within the hydrogel (3D approach).

## What are hydrogels?

Hydrogels are polymeric networks that can absorb a large quantity of water or any fluid (up to 95–99% of their weight). They are biomimetic per se since their high water content and diffusive transport properties very closely resemble those of the natural ECM ^11^. The majority of hydrogels are also biocompatible, such as the ones based on natural polymers—agarose, alginate, chitosan, collagen, fibrin, gelatin, hyaluronic acid, dextran, silk, and matrigel ^12^—as well as synthetic gels based on poly(ethylene glycol) (PEG), poly(vinyl alcohol) (PVA), poly(propylene fumarate), polyacrylic acid (PAA), and poly(hydroxyethyl methacrylate) (PHEMA) ^13^. An additional characteristic of hydrogels, very useful in TE and regenerative medicine, is their high potential for functionalization, which renders them easy to customize for improved cell-adhesion options and mechanical properties or sustained release of growth factors, cytokines, and drugs ^14^.

There are different kinds of hydrogels: (a) physical or reversible gels, where the network of polymers is based on the physical cross-links such as micellar crystallites, helix formation, hydrogen bonding, or hydrophobic forces, which can be ‘broken’ (reversed) by changing of the conditions (pH, temperature, salt concentration and ionic strength of the solution) ^15^, and (b) chemical or permanent gels, where the cross-linked polymers are covalently bound. An important fact for cartilage TE is that hydrogels possess tunable mechanical properties, which are related to the degree of cross-linking and affected by the presence and amount of charge. Charged hydrogels undergo changes in swelling related to pH status and in shape if exposed to the electromagnetic field ^16^. A very interesting recent study by Offeddu *et al*. investigated the difference in mechanical response and morphology of physically cross-linked PVA and PAA cryogels (structures obtained by repeated freezing and thawing of the polymer solution) versus heat-treated chemical gels made from the same polymers, as a result of pH-dependent swelling ^17^. The elastic modulus of the physical cryogels, in contrast to that of the heat-treated chemically cross-linked gels, was found to increase with charge activation and swelling and this was explained by the occurrence of electrostatic stiffening of the polymer chains at large charge densities ^17^. This is one of the first studies to report such cartilage-like mechanical behavior of hydrogels. In this context, it is important to mention that induced hydrogel swelling can also destabilize immature collagen in the newly engineered construct, preventing it from developing into the robust ECM-like collagen framework characteristic of native cartilage. The Vunjak-Novakovic, Hung, and Ateshian group hypothesized that this problem can be overcome by mechanically constraining the tissue swelling ^18^. To this aim, they developed a novel constraint-based culture system—a ‘cage’ which enhanced collagen maturation through the increased formation of pyridinoline cross-links and improved collagen matrix stability.

## Gradient hydrogels

A broad definition of a ‘gradient hydrogel’ is a hydrogel possessing a gradual and continuous spatiotemporal change in at least one property ^19^. Gradient hydrogels are excellent tools for engineering the native-like, biomimetic, cellular microenvironment. They also allow analyses of a wide spectrum of property values in a single sample and this is well suited for high-throughput screening ^20^. Gradients can be physical or biochemical or a combination of the two and have a temporal component as well. Gradient hydrogels can be generated via various methods that usually involve inducing cross-linking of the precursor solution, such as photopolymerization, the enzyme-catalyzed method, and temperature-induced gradient ^12^. To achieve gradient via photopolymerization, a photomask is used to block the ultraviolet (UV) light and subsequently prevent cross-linking on the covered region of the gel. The mask can be stationary or sliding. With the latter, first the whole polymer gets exposed to UV light and then the mask gradually covers the entire gel, moving in a linear fashion from one end. For example, if the cross-linking increases stiffness, the first covered hydrogel region will be most elastic while the last covered region, which was exposed to UV light for the longest time, undergoes a higher degree of cross-linking and is most stiff. In this way, the stiffness gradient is achieved ^21^. Enzymatic preparation of hydrogels comprises the use of enzyme systems such as tyrosinases, transferases, and peroxidases that catalyze covalent cross-linking of hydrogel precursors. These reactions can be performed *in situ*—where the gel precursors with bioactive substance are administered via a syringe locally, at the site where the gelation should be induced (for example, at the wound site)—and are particularly useful for generating dynamic scaffolds and systems for controlled release ^22^. Enzyme-catalyzed mild cross-linking has several advantages over UV-mediated photopolymerization, such as its very low cytotoxicity. Low toxicity is due to the fact that majority of the used enzymes are naturally occurring and the reactions are catalyzed at physiological conditions—neutral pH, moderate temperatures, in aqueous solution. Enzymatic gelation can also be combined with light-triggered chemical immobilization to achieve hydrogel patterning and gradient ^23^. This can be performed *in situ* as well. Mosiewicz *et al*., from the Lutolf group, describe how they achieved highly localized spatial patterning and tethering of a biomolecule of interest ^24^. They induced photosensitivity of a peptide substrate of activated transglutaminase factor XIII (FXIIIa), a key ECM cross-linking enzyme, by masking its active site with a photolabile cage group. Such a caged, inactive enzymatic peptide substrate was covalently incorporated into a PEG hydrogel. Localized cleavage of the cage and reactivation of the enzyme substrate were achieved by controlled light exposure from a confocal laser. Subsequent FXIIIa reaction of the uncaged substrate with a counter-reactive substrate on a biomolecule of interest allowed covalent biomolecule tethering in a highly localized, user-defined pattern ^24^.

The gradient in nanocomposite hydrogels (hydrogels with integrated nanomaterials characterized by higher elasticity and strength in comparison with traditional hydrogels ^25, 26^) can be induced before the cross-linking, by the application of electric or magnetic field. Microfluidics, additive manufacturing (AM), and microsphere-based approaches are also very efficient methods of generating gradients. A number of excellent reviews give a detailed overview of methods to generate gradient hydrogels in view of their relevance to TE in general ^1, 20, 27^. Here, we focus on gradient hydrogels for cartilage TE and give representative references to the innovative approaches used for their fabrication and implementation.

### Physical gradients

Physical gradients are mechanical gradients (variations in stiffness—that is, Young’s modulus values), density, strain/stress, and porosity/pore size gradients. Recently, AM techniques gained popularity for the fabrication of physical gradients. These include stereolithography, fused deposition modeling, selective laser sintering, inkjet, and extrusion bioprinting ^28, 29^. A very good review by Bracaglia *et al*. provides details on each of the AM methods as well as methods for the evaluation of established physical gradients such as micro-computed tomography ^28^.

Zhu *et al*. proposed a hypothesis that stiffness gradient can induce a zonal-specific response of encapsulated cells in 3D, where the newly deposited tissues in gradient hydrogels will mimic the zonal organization of native articular cartilage ^30^. To test this hypothesis, they used a tissue-scale, photo-cross-linkable, multi-arm PEG hydrogel system as a backbone and chondroitin sulfate methacrylate, mixed with two cell-containing precursor solutions: neonatal bovine chondrocytes or human mesenchymal stromal cells (hMSCs). In this way, they fabricated 3D gradient hydrogels divided into zones 1–5 by the increasing stiffness ranging from 2 to 60 kPa, obtained by using two precursor solutions containing 2% (wt/vol) and 20% (wt/vol) PEG. The results showed that chondrocytes encapsulated in gradient hydrogels exhibited a native-like ECM distribution, and larger nodules of type II collagen were observed in zone 1 and larger sGAG nodules in zone 5. The chondrocytes were more hypertrophic as the stiffness increased from zone 1 to zone 5. Increasing matrix stiffness led to a stiffness-dependent increase in cartilage-specific gene expression by hMSCs, and there was a two- to three-fold higher expression of the genes encoding aggrecan and type II collagen in the stiffer zone (zone 5) compared with the softer zone (zone 1) ^30^. More details on the gradient stiffness hydrogel fabrication methods and mechanical property tests can be found in a recent review by Xia *et al*. ^31^.

Ren *et al*. also engineered zonal cartilage by bioprinting collagen type II hydrogel constructs with a biomimetic cell density gradient ^32^. They bioprinted two types of hydrogel/cell construct: one with a biomimetic chondrocyte density gradient—the ratio of chondrocyte densities in the superficial zone (top 10%) to the transitional zone (middle 10%) to the deep zone (remaining 80%) of normal adult human articular cartilage is approximately 3:2:1, which the authors achieved in their constructs, and the ratio of the three zonal volumes was 13%:13%:74%—and the other type with a homogenous cell density, equal to the total cell density of 1 × 10 ^7^ cells/mL. This amount was chosen since this is the total cell density within the articular cartilage of the human medial femoral condyle ^33^. As a result, the cell density gradient distribution was reflected in a zonal ECM distribution.

***Porosity.*** Even though most hydrogels that are used for TE and cell encapsulation facilitate diffusive transport of oxygen and many nutrients, and thus do not need pores, pores can offer the ability to fabricate hydrogels first and then seed them with cells ^26^. Porous hydrogels can be fabricated in several ways: (i) by making the gel network with encased biodegradable blocks ^34^, (ii) by making hydrogel fibers via electrospinning or 3D bioprinting ^28, 35^, (iii) through the application of porogens such as polymer microspheres ^36, 37^, or (iv) with the 3D laser perforation ^38^. Under mechanical loading, a strain gradient also exists in hydrogel scaffolds; the superficial hydrogel layer absorbs more strain than the middle and deep layers ^39^.

A combination of methods is frequently implemented for making physical gradients, particularly for composite hydrogels. Su *et al.* used an electrophoresis method to induce hydroxyapatite (HA) nanoparticles within the matrix of PVA hydrogels prepared by a directional freezing-thawing (DFT) process. By controlling the time of the electrophoresis process, they obtained a bilayered gradient hydrogel containing HA particles in only half of the gel region. The PVA/HA composite hydrogel exhibited physical gradient (mechanical strength) depending on the distance to the cathode ^40, 41^. PVA/HA combination yields multilayered composite material with rheological properties similar to those of natural cartilage ^41^. This method holds promise to be more efficient than mentioned above for generation of physical gradients.

Physical gradients are very important when engineering the OC interface, which is the junction between the cartilaginous and bone phase. Cross *et al*. report a gradient scaffold with two natural polymers—gelatin methacryloyl (GelMA) and methacrylated kappa carrageenan (MκCA)—reinforced with 2D nanosilicates to mimic the native tissue interface ^42^. The addition of nanosilicates results in shear-thinning characteristics of prepolymer solution and increases the mechanical stiffness of cross-linked gradient structure. A gradient in mechanical properties, microstructures, and cell adhesion characteristics was obtained by using a microengineered flow channel ^42^. They also achieved cell morphology gradient since the hMSCs encapsulated in the MκCA and GelMA hydrogels gained chondrocyte- and osteoblast-like morphology, respectively. At the interface regions, both of the cell morphologies were present. Such a structure not only can effectively mimic the native interface but also can provide a seamless connection between the cartilage and bone phase of the whole OC construct.

Recreating the physical microenvironment as well as cell-matrix interactions is crucial during the differentiation of mesenchymal progenitors. In this context, it is important to mention the work of the Burdick group on the ‘HAVDI’ hydrogels (that is, the methacrylated hyaluronic acid [MeHA] hydrogel system that, across a physiological range of ECM stiffness, enables the independent co-presentation of the HAVDI adhesive motif from the EC1 domain of N-cadherin and the RGD adhesive motif from fibronectin) ^43^. The HAVDI hydrogels are particularly interesting since they also provide specific cell-material interactions; namely, they give the option for the mesenchymal stem/stromal cells to interact with the MeHA backbone polymer through several cell surface receptors that are expressed by MSCs, including CD44 and CD168 ^44^.

Another aspect of cell-matrix interactions which needs to be recapitulated in the engineered construct is the cell-triggered proteolysis of the ECM, which is mediated by the members of the metalloproteinase (MMP) family ^45^. The Lutolf group engineered synthetic PEG-based hydrogels with a combination of cross-linking MMP substrates, as linkers between PEG chains and cell adhesion oligopeptide ligands (RGDSP), and showed *in vitro* and *in vivo* in the models for bone regeneration that such hydrogels can undergo cell-mediated proteolytic degradation followed by the remodeling into a cell-secreted bone matrix at the site of the injury ^46^.

### Biochemical gradients

Biochemical gradients are gradients in concentration of the bioactive molecules—morphogens (growth and transcription factors, chemokines, and cytokines). As mentioned earlier, biocompatible hydrogels based on natural and some synthetic polymers possess inherent bioactive properties, which can be enhanced through functionalization, such as covalent binding of peptides and proteins ^47^ or exopolysaccharides ^48^ to the hydrogel polymers, or with additional affinity binding achieved by adding, for example, collagen binding sequences to the peptide/protein to be incorporated into the hydrogel ^49^. Synthetic hydrogels are usually functionalized in the direction of achieving better cell adhesion by introducing cell-adhesive ligands such as RGD(S) peptide, the key component of the cell attachment domain of fibronectin ^7^.

Functionalization can also provide temporal gradients—time-specific presentation of bioactive sequences/molecules. In an innovative study, Parmar *et al*. used MMP7-functionalized recombinant bacterial collagens—streptococcal collagen-like 2 (Scl2) proteins—to enable temporal presentation of RGDS peptide and fine-tune the chondrogenic differentiation of hMSCs ^50^. The RGDS binding sites were sequentially enzymatically released from the hydrogel via the MMP7-cleavable peptides used for functionalization ^50^. The rationale was based on previous studies that the persistence of the RGD peptide expression can delay or alter chondrogenic differentiation of hMSCs, often leading to hypertrophy ^51^.

Gradients of growth factors are among the crucial components in biomimetic cartilage TE. The key growth factors are the members of the transforming growth factor-beta (TGF-β) superfamily (including bone morphogenetic proteins [BMPs] ^52^ and growth and differentiation factors ^53^), fibroblast growth factor (FGF) family, and insulin-like growth factor-I (IGF-I). During *in vivo* chondrogenesis, these factors are expressed in a time-dependent manner ^6^. This can be simulated *in vitro* by the sequential application to the cell culture—for example, basic FGF or FGF2 followed by BMP2 or IGF1, TGFβ2, or TGFβ3 ^6, 54^—or through the growth factor gradient in the hydrogel scaffold. One of the hallmark studies in establishing growth factor gradients in hydrogel scaffolds for OC engineering is by Wang *et al*., who used microsphere-mediated growth factor delivery in polymer scaffolds and evaluated the impact on OC differentiation of hMSCs ^55^. They used two recombinant human growth factors (rhBMP-2 and rhIGF-I) and tested delivery efficiency of polylactic-co-glycolic acid (PLGA) and silk fibroin microspheres incorporated as gradients into an alginate hydrogel. Both growth factors formed deep and linear concentration gradients in the scaffold directing hMSCs to osteogenic and chondrogenic differentiation along the concentration gradients, which led to formation of zonality in the engineered construct ^55^. A very good recent review by Gupta *et al*. ^36^ provides more details on the microsphere-based scaffolds that followed after the described study ^56^.

In addition to microsphere-based methods for generating biochemical gradients, methods such as layer-by-layer, prepolymer mixing, or modular assembly are tested, and AM methods are gaining ground in generating biochemical gradients, similarly as for the physical gradients ^28^. The microfluidic-based generators with precise fluid control are also implemented, as either mono-phase or droplet-based methods ^57^. A detailed review by Samorezov and Alsberg provides more information on strategies and methods for spatiotemporal control over bioactive factor delivery, concerning both generating patterns of bioactive factors on scaffold surfaces and building up patterned 3D scaffolds ^58^.

### Dual gradients of mechanical and biochemical cues

SCs are being increasingly used in cartilage TE ^59^. To direct SC differentiation or influence SC production of the autocrine/paracrine factors or both, one needs to provide a niche effect—a microenvironment with multiple biochemical and physical factors. To this aim, Tong *et al*. describe a multi-arm PEG-based gradient hydrogel platform as a biomimetic cell niche containing independently tunable matrix stiffness and biochemical ligand (CRGDS peptide) density ^60^. They introduced both gradients—first the mechanical one and then the chemical one—using a gradient of UV exposure over the precursor solution and over hydrogels with established mechanical gradients, respectively ^60^. This system is intended for general use and is not focused on cartilage. Tam *et al*. created a biomimetic 3D cell culture with dual biochemical and physical gradients by using photosensitive agarose and hyaluronic acid hydrogels that are activated by single- or two-photon irradiation for biomolecule immobilization at specific volumes within the 3D hydrogel ^61^. This platform has been optimized for modeling the nervous system and cancer ^61^. Other attempts to provide dual biochemical and physical gradients have been made by using microfluidic platforms ^62^. However, the micro-scale platforms are not well suited for establishing gradient hydrogels in clinically relevant tissue constructs. Up to now, the most promising approach for generating dual large-scale (that is, tissue-scale) gradient hydrogels in the context of cartilage TE is the method (described above) by Zhu *et al*. ^30^.

## Future challenges

Fabrication of gradient hydrogels has significantly advanced in recent years. However, a number of challenges are still associated with their use as one of the components in biomimetic cartilage TE. This is especially true concerning the potential clinical application, which would require large-scale constructs with fully native-like zonal composition, structure, and functions. Even though many of the described methods can already create centimeter-scale implants with gradients (for example, layered hydrogels, grayscale masks with photopolymerization, and porosity), it is still not possible to completely mimic the natural zonality and microenvironment of the articular cartilage ^63^. Another aspect to consider is to specify the best hydrogel degradation parameters since it is still challenging to match the degradation of hydrogels with the growth of cartilage and OC tissues ^64^.

Other challenges include methods for establishing more efficient temporal gradients that would provide more native-like gene expression during engineered cartilage formation, which is still an issue as evidenced by the lack of a mature chondrocyte-like phenotype in the hMSC-engineered constructs ^6^. Good tools to further investigate the response of SCs to lineage-guiding metabolites are the tunable supramolecular hydrogels. Supramolecular hydrogels are macromolecular polymers stabilized by noncovalent bonds (for example, hydrogen bond, hydrophobic interaction cation–π, and π–π interactions) into a solid 3D network ^65^. The Ulijn group generated supramolecular hydrogels composed of fibers with cytocompatible surface functionality and tunable network densities that can help in selection of bioactive metabolites that can specifically target bone and cartilage formation ^66, 67^.

We still need to develop efficient ways of generating gradients of other important bioactive molecules, such as oxygen tension gradient ^68^ and gradients of insulin, ascorbate, and glucose ^69^. Another interesting aspect is establishing gradients of ‘raw materials’ such as chondroitin sulfate incorporated into the hydrogel ^70– 72^. Also, more work needs to be done on control of the localized molecular orientation, such as different alignments of collagen fibers in cartilage zones ^16^.

In conclusion, physical patterning is not sufficient to recreate the zones, and the combination of physical and chemical cues as well as a more sophisticated understanding of the biology will be needed to create successful gradients for cartilage TE.

## Abbreviations

AM, additive manufacturing; BMP, bone morphogenetic protein; ECM, extracellular matrix; FGF, fibroblast growth factor; FXIIIa, activated transglutaminase factor XIII; GelMA, gelatin methacryloyl; HA, hydroxyapatite; HAVDI, His-Ala-Val-Asp-Lle; hMSC, human mesenchymal stromal cell; IGF-I, insulin-like growth factor-I; MκCA, methacrylated kappa carrageenan; MeHA, methacrylated hyaluronic acid; MMP, metalloproteinase; MSC, mesenchymal stromal cell; OC, osteochondral; PAA, polyacrylic acid; PEG, poly(ethylene glycol); PVA, poly(vinyl alcohol); RGD, Arg-Gly-Asp; RGDS, Arg-Gly-Asp-Ser; SC, stem cell; sGAG, sulphated glycosaminoglycan; TE, tissue engineering; TGF-β, transforming growth factor-beta; UV, ultraviolet.
